# Paracetamol toxicity in classic homocystinuria: Effect of *N*‐acetylcysteine on total homocysteine

**DOI:** 10.1002/jmd2.12363

**Published:** 2023-03-03

**Authors:** Nour Elkhateeb, Sarah Hyde, Sarah L. Hogg, Daniel Allsop, Arun Shankar, Patrick Deegan, Chong Y. Tan

**Affiliations:** ^1^ Department of Metabolic Medicine Cambridge University hospitals NHS Foundation Trust Cambridge UK; ^2^ Department of Clinical Genetics Cambridge University hospitals NHS Foundation Trust Cambridge UK; ^3^ Department of Gastroenterology Norfolk and Norwich University Hospital NHS Trust Norwich UK; ^4^ Biochemical Genetics Unit Cambridge University Hospitals NHS Foundation Trust Cambridge UK; ^5^ Department of Histopathology Norfolk and Norwich University Hospitals NHS Trust Norwich UK

**Keywords:** acute liver failure, classical homocystinuria, glutathione depletion, *N*‐acetylcysteine, paracetamol toxicity, plasma total homocysteine

## Abstract

Classical homocystinuria (HCU) is caused by cystathionine β‐synthase deficiency leading to impaired homocysteine transsulfuration and accumulation of homocysteine and methionine. Patients present with a wide spectrum of manifestations including ocular, skeletal, neuropsychiatric, and vascular manifestations. We report a 48‐year‐old female with pyridoxine‐unresponsive HCU treated with betaine, cyanocobalamin, and folate. Her diet was non‐restricted due to intolerance of low‐methionine diet. She was admitted to hospital following a fall, with multiple fractures and subsequently developed acute liver failure with encephalopathy. Shock, sepsis, and liver ischaemia/thrombosis were excluded. In the context of glutathione depletion expected in HCU, hepatic dysfunction was presumed to be due to iatrogenic paracetamol toxicity, despite paracetamol intake at conventional therapeutic dose, with role of hypermethioninemia as a contributing factor being uncertain. Betaine was discontinued on hospital admission. *N*‐Acetylcysteine (NAC) infusion was initiated. Plasma total homocysteine (tHcy) was 3.4 μmol/L 9 days following initiation of NAC treatment with a markedly elevated plasma methionine of 1278 μmol/L. tHcy concentration returned to pre‐admission baseline after NAC was discontinued. Recovery following this episode was slow with a prolonged cholestatic phase and gradual improvement in jaundice and coagulopathy. We recommend that paracetamol should be administered cautiously in HCU patients due to underlying glutathione depletion and risk of toxicity even at therapeutic doses. NAC is clearly effective in lowering tHcy in classical HCU in the short‐term however further research is required to assess clinical efficacy and use as a potential therapy in classical HCU.

**SYNOPSIS**

Patients with classical homocystinuria are at risk of acute liver failure due to paracetamol toxicity that may develop even at therapeutic doses. *N*‐Acetylcysteine is effective in lowering total plasma homocysteine in classical homocystinuria in the short‐term.

## 1 INTRODUCTION

Classical homocystinuria (HCU) is an inborn error of metabolism caused by cystathionine β‐synthase (CBS) deficiency leading to impaired catabolism of the sulphur containing amino acid, methionine, through the transsulfuration pathway and subsequent accumulation of homocysteine (Hcy) and methionine, and reduced levels of cystathionine, cysteine, or cystine (disulphide of the cysteine) in blood.^1^

Patients with HCU are divided into pyridoxine non‐responders, partial, full and extreme responders based on the response of plasma total homocysteine (tHcy) to pharmacological doses of pyridoxine.^2^ Pyridoxal 5‐phosphate, the active form of pyridoxine, binds the catalytic domain of CBS.^3^

Treatment of pyridoxine non‐responsive HCU includes dietary intervention with low natural protein, and supplements of a methionine‐free L‐amino acid mixture,^4^, ^5^, ^6^ betaine,^7^, ^8^ folate, and vitamin B12.^1^

Patients present with a wide spectrum of manifestations, ranging between early onset severe multi‐system disease to a mild asymptomatic state. Clinical manifestations include ocular, skeletal, neuropsychiatric, and vascular manifestations, with a milder phenotype observed in pyridoxine‐responsive patients,^1^, ^9^ and possible asymptomatic CBS deficiency found in some individuals who are homozygous for the *CBS* c.833T>C (p.Ile278Thr) variant.^10^

In the current study, we report an adult patient with pyridoxine non‐responsive HCU on betaine therapy who developed acute liver failure in the context of paracetamol toxicity, with markedly elevated methionine. We report the potential therapeutic role of *N*‐acetylcysteine (NAC) in lowering plasma tHcy in HCU.

## 2 CASE REPORT

We report a 48‐year‐old female with a diagnosis of pyridoxine‐unresponsive HCU. The diagnosis was established at age 2 years following lens subluxation and biochemical confirmation. The patient developed several complications of HCU including learning difficulties, behavioural and mood issues, severe visual impairment (left dislocated lens and right aphakia following vitrectomy with lens extraction for lens subluxation), skeletal complications (osteoporosis, scoliosis and history of tibial fracture), vascular parkinsonism and thrombotic complications (deep vein thrombosis [DVT] in left leg at age 46 years). She remained on a non‐restricted diet, due to intolerance of the special diet, and treatment with betaine 6 g three times daily (TDS), cyanocobalamin 50 mcg daily and folic acid 5 mg daily. She continued on anticoagulation with direct‐acting oral anticoagulant (rivaroxaban 20 mg daily) since DVT diagnosis.

At age 48 years, she sustained an unwitnessed fall, presumably secondary to her visual impairment. A trauma sequence CT scan identified a moderate left pneumothorax with extensive left rib fractures creating a flail segment, multiple fractures of the spinal transverse processes and a left gleno‐humeral fracture. A left sided chest drain was inserted. Blood tests on admission demonstrated normal kidney function and standard liver blood tests showed a total bilirubin (Tbil) of 22 μmol/L, serum alanine transaminase (ALT) 59 U/L, and serum albumin 33 g/L (Table 1). Conservative management with analgesia and left collar and sling was advised for the multiple fractures. The patient was started on serratus plane block, a fentanyl patient‐controlled analgesia, gabapentin 100 mg TDS and paracetamol 1 g four times daily orally or intravenously (iv) if not tolerated orally. Oral betaine was withheld on admission as the patient was nauseated and vomited intermittently and had reduced oral intake since admission. Her weight on admission was 66.8 kg. Her weight on Day 7 on admission was recorded as 57 kg (change −9.8 kg/14.6%).

**TABLE 1:** Biochemical abnormalities and treatment details before and during admission.

	Pre‐admission baseline	Day 0 admission	Day 10 admission (onset of ALF)	Day 20 admission	Day 47 admission	Day 58 admission	Day 72 admission	Day 97 (discharge)
Treatment	CNCbl Betaine Unrestricted diet	Betaine held	NAC started Betaine held	On NAC Betaine held	NAC stopped 11 days before Betaine held	Betaine held	Betaine held	CNCbl Unrestricted diet resumed Betaine held
tHcy (μmol/L)	67.2	N/A	N/A	3.4	64.6	65.7	54.3	N/A
Plasma methionine (μmol/L)	640	N/A	N/A	1278	1410	1143	512	N/A
ALT (U/L)	31	59	3789	313	65	40	23	40
ALP (U/L)	46	51	240	240	216	174	146	132
GGT (U/L)	N/A	N/A	488	N/A	N/A	68	N/A	N/A
Tbil (μmol/L)	11	22	129	274	329	228	98	57
Alb (g/L)	33	33	23	14	39	38	27	21
INR	N/A	N/A	6.08	2.15	1.8	2.6	1.43	N/A
CRP (mg/L)	N/A	2	18	N/A	N/A	N/A	N/A	N/A
Plasma lactate (mmol/L)	N/A	N/A	6	N/A	N/A	N/A	N/A	N/A

*Note*: Reference values: tHhcy: 0.0–16.0 μmol/L, plasma methionine: 17–37 μmol/L, Tbil: <21 μmol/L, ALT: 0–45 U/L, Alb: 38–55 g/L, ALP: 30–120 U/L, GGT: 0–45 U/L, CRP: 0–5 mg/L, serum lactate: 0.5–2.2 mmol/L.

Abbreviations: Alb, serum albumin; ALF, acute liver failure; ALP, alkaline phosphatase; ALT, alanine transaminase; CNCbl, cyanocobalamin; CRP, C‐reactive protein; GGT, gamma‐glutamyl transferase; INR, international normalised ratio; NAC, *N*‐acetylcysteine; Tbil, total bilirubin; tHcy, total plasma homocysteine.

Ten days after admission she became severely hypoglycaemic with an unrecordable blood glucose concentration that was corrected with oral fast acting dextrose gel, glucagon intramuscularly and 500 mL 10% iv dextrose. She had an episode of large volume coffee ground vomiting. Blood tests at that time were consistent with acute liver failure (ALT 3789 U/L, serum alkaline phosphatase [ALP] 240 U/L, Tbil 129 μmol/L) with elevated plasma lactate and CRP with normal kidney function (Table 1), plasma ammonia was 118 μmol/L (and subsequently dropped to 87 μmol/L; reference range 11–30 μmol/L) which was attributed to acute liver failure. She was drowsy with Grade II hepatic encephalopathy and a hepatic flap. Neuroimaging was not done at that time. The working diagnosis was acute liver failure secondary to paracetamol toxicity. The patient received 26 g of paracetamol in the 240 h after her first dose of paracetamol on hospital admission, equating to an average of 2.6 g per 24‐h period. She never received more than 4 g paracetamol in any one 24‐h period. Plasma paracetamol concentration was not measured at that time.

The patient was transferred to the intensive care unit (ICU), given iv vitamin K and started NAC at a dose of 8.4 g over 1 h, 2.8 g over 4 h, and then 5.5 g over 16 h. She was also started on broad spectrum antibiotics and antifungals to cover for infection. An urgent triple phase liver CT scan demonstrated patent hepatic vasculature and excluded hepatic ischemia as the cause of acute liver failure. Viral, autoimmune hepatitis screening, and Wilson disease screening were negative. Rivaroxaban was discontinued due to the coagulopathy. She continued NAC at 5.5 g per 16 h, daily for a further 26 days.

Biochemical testing at 10 days following onset of acute liver failure showed a plasma tHcy of 3.4 μmol/L and a markedly elevated plasma methionine of 1278 μmol/L. By that time, she had returned to her baseline neurological status and did not show clinical features of brain oedema, increased intracranial pressure or seizures. After NAC was discontinued for 9 days, plasma tHcy increased to 64.6 μmol/L, comparable to her pre‐admission baseline, and plasma methionine peaked at 1410 μmol/L, which gradually recovered to 512 μmol/L prior to discharge, comparable to her baseline levels.

**FIGURE 1:**
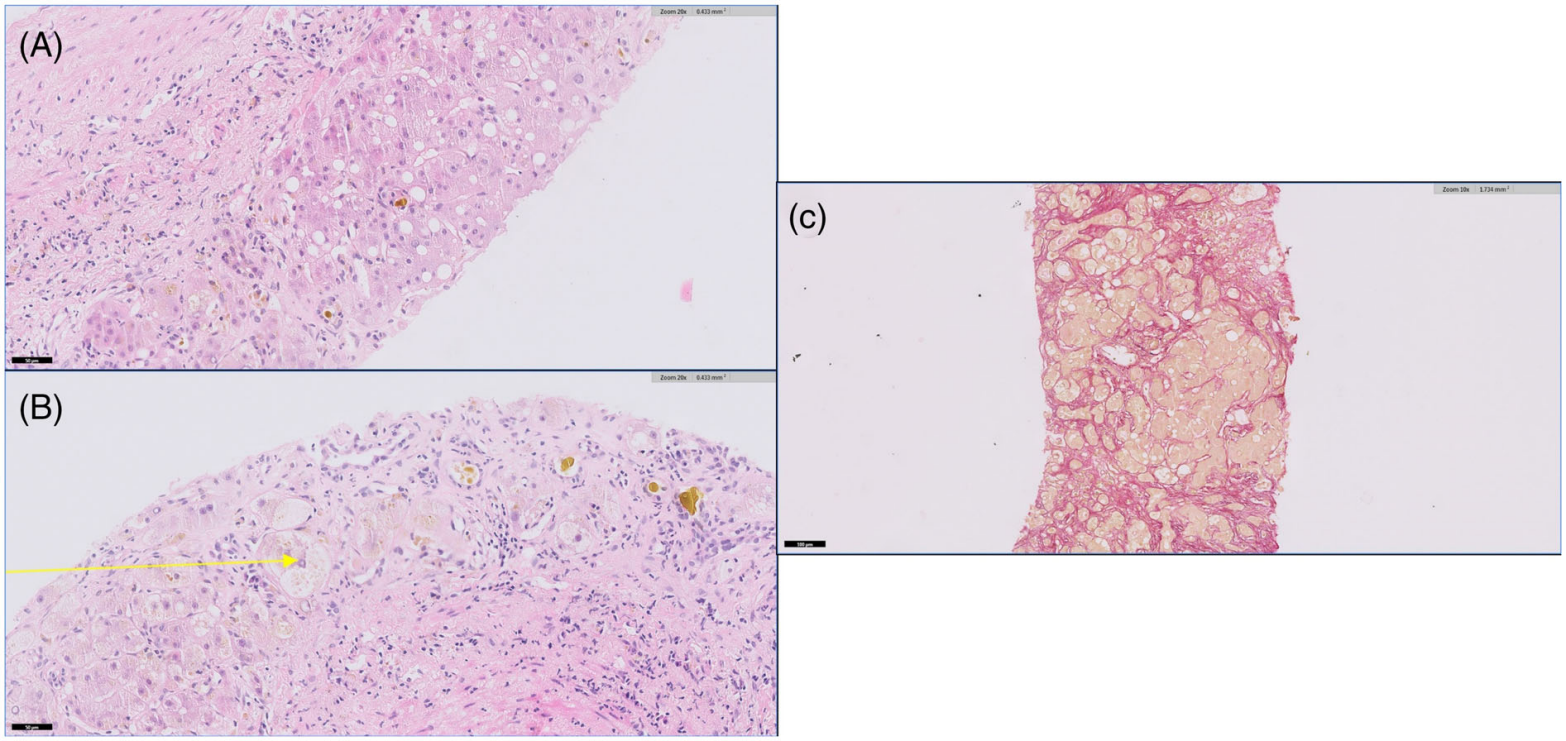
Histopathological abnormalities in the liver biopsy. (A) Haematoxylin and eosin‐stained liver core showing steatosis with marked cholestasis. (B) Haematoxylin and eosin‐stained liver core showing a ballooned hepatocyte (yellow arrow) with scattered necro‐inflammatory foci in keeping with steatohepatitis. (C) Elastic Picrosirius Red‐stained liver core showing advanced fibrosis.

Hepatic recovery following this episode of acute liver failure was slow with a prolonged cholestatic phase and gradual improvement in jaundice and coagulopathy (Table 1). There were concerns regarding possible post‐necrotic cirrhosis; a liver biopsy showed features of steatohepatitis with macrovesicular steatosis, necroinflammation, cholestasis and advanced acute/subacute fibrosis. This was associated with inconspicuous inflammatory infiltrate with prominent plasma cells (Figure 1).

Recovery was also complicated by pulmonary oedema secondary to hypoalbuminemia; ileus secondary to hypokalaemia; feeding intolerance and vomiting with a long period of nasojeujenal and nasogastric feeding. She required several blood transfusions for epistaxis secondary to coagulopathy. The patient was discharged following an admission of 97 days including 14 days in ICU. On discharge, she was fully conscious and tolerating oral intake. She continues on unrestricted diet (as the patient declined to commence on a low‐methionine diet or resume betaine treatment) in addition to folic acid and cyanocobalamin. Anticoagulation was resumed after resolution of coagulopathy. She is not receiving betaine. Her ALT is within the normal range while bilirubin has marginally increased post‐discharge. Post‐discharge plasma tHcy and methionine remain elevated (Table 1). Plasma cysteine or cystine concentrations were not measured in the past or during the admission.

## 3 DISCUSSION

We describe an adult patient with pyridoxine non‐responsive HCU on betaine treatment who presented with acute liver failure in the context of presumed iatrogenic paracetamol toxicity. Intravenous NAC has reduced plasma tHcy in the patient.

Acute liver failure has been previously reported in patients with HCU. In a previous report, an 18‐year‐old patient with poorly controlled HCU (who was on betaine treatment) developed acute liver failure after paracetamol intake, with a paracetamol level not suggestive of overdose of paracetamol. Plasma methionine was markedly raised up to 1366 μmol/L. The patient showed complete recovery of liver function.^11^ This was a similar presentation to our case, where acute liver failure developed because of presumed paracetamol toxicity with a conventionally therapeutic dose of paracetamol. Another report reported a 31‐year‐old patient with late‐treated HCU (treatment started at age 22 years) who developed liver cirrhosis and chronic liver failure and required liver transplantation.^12^ Liver abnormalities have been reported in CBS‐knockout mice including enlarged hepatocytes with microvesicular lipid droplets,^13^ inflammation, fibrosis, and hepatic steatosis with evidence of oxidative stress with subsequent mitochondrial damage leading to liver injury.^14^ These changes are similar to the ones reported in our patient's liver biopsy.

Cysteine is a semi‐essential amino acid that is essential for synthesis of glutathione (GSH),^15^ which is a tripeptide (glutamate–cysteine–glycine) and an antioxidant with a key role in cellular protection from endogenous and exogenous reactive oxygen species (ROS) and is essential for mitochondrial function and maintenance of mitochondrial DNA.^16^ Decreased concentrations of plasma cysteine and liver GSH were shown in CBS‐deficient mice despite adequate dietary cysteine intake.^17^ In addition, disturbed GSH metabolism with significantly decreased total and reduced GSH and increased oxidised GSH was reported in patients with combined methylmalonic aciduria with HCU due to Cobalamin C defect.^18^ Thus, disturbed redox homeostasis and subsequent oxidative stress is implicated in the pathophysiology of HCU, which has shown by in vivo and in vitro studies.^19^, ^20^

Betaine is a methyl donor for betaine‐homocysteine S‐methyltransferase enzyme which catalyses the remethylation of Hcy to methionine in the liver. It is used in treatment of HCU, yet it does not fully correct the level of tHcy in HCU patients and increases plasma methionine level.^8^, ^21^ Cerebral oedema and hypermethioninaemia encephalopathy with reversible neuroimaging abnormalities have been reported in patients on betaine therapy with plasma methionine levels close or exceeding 1000 μmol/L.^21^, ^22^, ^23^ Plasma methionine levels have been reported to range from normal to extremely high in patients with fulminant hepatic failure, due to disturbed methionine metabolism.^24^, ^25^

We report elevated concentrations of methionine (>1000 μmol/L) during ICU admission in our patient, despite betaine having been stopped on admission. This could be attributed to disturbed methionine metabolism due to liver failure. Hypermethioninaemia encephalopathy may have been a contributing factor to patient's initial encephalopathy on ICU admission, in addition to hepatic encephalopathy. However, the encephalopathy resolved quickly despite persistently elevated plasma methionine (>1000 μmol/L). Neuroimaging to identify abnormalities associated with hypermethioninaemia encephalopathy was not done.

Patients with inherited methylation disorders associated with elevated plasma methionine (glycine N‐methyltransferase deficiency, S‐adenosylhomocysteine hydrolase deficiency, adenosine kinase deficiency) have been reported to develop liver abnormalities with abnormal liver histology, transaminitis, hepatosteatosis, and fibrosis.^26^, ^27^, ^28^ Animal studies showed that excess methionine induces hepatoxicity by binding hepatic ATP to form S‐adenosylmethionine and S‐adenosylhomocysteine with subsequent hepatic ATP depletion. This also results in shift in hepatic reduced GSH to oxidised GSH with subsequent oxidative damage.^29^, ^30^ The role of hypermethioninaemia in development of liver abnormalities in these disorders is uncertain.^26^, ^27^ It is not clear whether hypermethioninaemia associated with CBS deficiency, protein‐unrestricted diet and betaine therapy may have contributed to or precipitated liver damage caused by paracetamol toxicity in this patient.

Paracetamol is a commonly used analgesic that is usually safe at doses used therapeutically. At high doses, it causes potentially fatal hepatotoxicity with associated centrilobular hepatic necrosis.^31^ Increased risk of toxicity occurs with advancing age, malnutrition, and acute high doses.^32^ Paracetamol is metabolised to *N*‐acetyl‐*p*‐benzoquinone imine (NAPBQI) by cytochrome P‐450. NAPBQI is detoxified at non‐toxic doses by GSH into an acetaminophen‐GSH conjugate. However, at toxic doses, hepatic GSH may be depleted by as much as 80%–90%,^33^ leading to covalent binding of NAPBQI to proteins in hepatocytes with subsequent hepatic necrosis.^32^ In addition, GSH depletion leads to increased oxidative stress due to decreased detoxification of ROS, with alteration in calcium metabolism, initiation of signal transduction responses and mitochondrial dysfunction. This further contributes to hepatic necrosis.^31^ We suggest that disturbed GSH metabolism and subsequent oxidative stress due to poorly controlled HCU in our patient predisposed them to hepatic toxicity from a conventionally therapeutic dose of paracetamol. This may have been exacerbated by recent weight loss during admission.

NAC has long been used as an antidote in paracetamol‐induced hepatotoxicity by restoring hepatic concentrations of GSH, through increasing plasma concentrations of cysteine. In addition, it has antioxidant effects which reduce oxidative stress implicated in paracetamol hepatotoxicity.^22^, ^34^ Hcy is 70%–80% bound to albumin, with the other two non‐albumin bound Hcy fractions being free reduced Hcy (1%–2%) and free oxidised Hcy (homocysteine, cysteine–homocysteine disulphide) (10%–20%).^35^ Oral or iv administration of NAC was found to rapidly reduce the levels of plasma tHcy by replacing Hcy in its binding to albumin (thiol exchange reaction) leading to the formation of mixed low‐molecular‐weight cysteine and NAC disulfides with high renal clearance and possibly increased metabolic bioavailability, thus enhancing its renal clearance.^36^, ^37^, ^38^, ^39^

In vivo and in vitro studies,^36^, ^37^, ^40^ have shown that NAC reduced tHcy by 20%–50% in a dose dependent fashion, while the free Hcy fraction was increased. Urinary excretion of total and free cysteine and Hcy was increased on NAC infusion.^37^ NAC supplementation in CBS‐deficient mice was shown to reduce tHcy and increase total cysteine compared to controls. However, total cysteine levels were not fully corrected in mice.^41^ These reports support our observations that tHcy in our patient was markedly reduced during NAC intravenous treatment for paracetamol toxicity and has returned to untreated baseline after cessation of infusion.

Although it is suggested that free Hcy generated from thiol exchange reactions will be eliminated by kidneys, the increase in free reduced Hcy may actually increase endothelial cell dysfunction,^38^, ^42^ as plasma free Hcy may more accurately represent the in vivo biological activity of Hcy.^42^ Furthermore, NAC treatment in CBS‐knockout mice model with hepatopathy decreased life span of pups, yet the CBS‐knockout mice failed to respond to betaine or exhibit several important features of the human HCU disease, thus the results from the CBS‐knockout mice model should treated with caution.^43^ These observations may raise caution for the use of NAC as a potential long‐term therapy in HCU.

To our knowledge, no studies exist in literature that described the potential therapeutic effect of NAC in patients with HCU. A phase I–II clinical cross‐over, unblinded, clinical trial studying the effect of paracetamol and NAC on liver metabolism in adult patients with HCU has been registered but recruitment was suspended due to COVID‐19 pandemic.^43^, ^44^ A non‐randomised open label, unblinded, clinical trial studying the effect of oral NAC in lowering homocysteine in individuals with HCU has been registered yet the results have not been deposited nor published in literature.^44^, ^45^

This report has limitations due to lack of data on plasma cysteine, cystine concentrations, and GSH metabolites. Long‐term monitoring of plasma tHcy and plasma amino acids on the patient's follow‐up is planned as per the published HCU guidelines.^1^

In conclusion, we suggest that paracetamol should be administered cautiously in HCU patients because of assumed GSH depletion and risk of toxicity even at therapeutic doses. Other risk factors for liver injury could possibly include hypermethioninemia due to betaine treatment. NAC administration is clearly effective in lowering tHcy in HCU on the short‐term, however further research is required to assess clinical efficacy and use as a potential therapy in HCU.

## AUTHOR CONTRIBUTIONS

Nour Elkhateeb and Sarah Hyde planned, wrote the report, which was then approved by all co‐authors. Sarah L. Hogg, Patrick Deegan, and Chong Y. Tan commented on the biochemical and metabolic aspects of the case. Arun Shankar commented on the hepatology aspects of the case. Daniel Allsop provided the figure and interpretation on histopathological liver abnormalities. All authors provided critical comments on the manuscript. All authors approved the submission.

## CONFLICT OF INTEREST STATEMENT

The authors declare no conflict of interest.

## ETHICS STATEMENT

A local Ethical Committee approval was not required. Informed consent to participate and publish was obtained. This article does not contain any studies with animal subjects performed by the any of the authors. All procedures followed were in accordance with the ethical standards of the responsible committee on human experimentation (institutional and national) and with the Helsinki Declaration of 1975, as revised in 2000. Informed consent was obtained from all patients for being included in the study. Proof that informed consent was obtained is available upon request.

## Data Availability

The authors confirm that the data supporting the findings of this study are available within the article [and/or] its supplementary materials.
